# The Incidence of Catatonia Diagnosis Among Pediatric Patients Discharged From General Hospitals in the United States: A Kids' Inpatient Database Study

**DOI:** 10.3389/fpsyt.2022.878173

**Published:** 2022-04-29

**Authors:** James Luccarelli, Mark Kalinich, Carlos Fernandez-Robles, Gregory Fricchione, Scott R. Beach

**Affiliations:** ^1^Department of Psychiatry, Massachusetts General Hospital, Boston, MA, United States; ^2^Department of Psychiatry, McLean Hospital, Belmont, MA, United States; ^3^Department of Psychiatry, Harvard Medical School, Boston, MA, United States

**Keywords:** catatonia, adolescent psychiatry, cohort studies, mood disorders, psychotic disorder, demography

## Abstract

**Objective:**

Catatonia is a neuropsychiatric condition occurring across the age spectrum and associated with great morbidity and mortality. While prospective cohorts have investigated catatonia incidence among psychiatric patients, no studies have comprehensively explored the incidence of catatonia in general hospitals. We examine the incidence of catatonia diagnosis, demographics of catatonia patients, comorbidities, and inpatient procedures utilized among pediatric patients hospitalized with catatonia in the United States.

**Methods:**

The Kids' Inpatient Database, a national all-payors sample of pediatric hospitalizations in general hospitals, was examined for the year 2019. Hospitalizations with a discharge diagnosis of catatonia were included in the analysis. Hospitalizations with catatonia as the primary discharge diagnosis were compared to hospitalizations with catatonia as a secondary discharge diagnosis.

**Results:**

A total of 900 (95% CI: 850–949) pediatric discharges (291 with catatonia as a primary diagnosis, 609 with catatonia as a secondary diagnosis) occurred during the study year. Mean age was 15.6 ± 2.6 years, and 9.9% were under age 13. Comorbidities were common among patients with catatonia, with psychotic disorders (165; 18.3%), major depressive disorder (69; 7.7%), bipolar disorder (39; 4.3%) and substance-related disorders (20; 2.2%) as the most common primary diagnoses. There was significant comorbidity with neurologic illness, developmental disorders, autism spectrum disorder, and inflammatory conditions. In total 390 catatonia discharges (43.3%) included at least one procedure during admission.

**Conclusions:**

catatonia is rarely diagnosed in pediatric patients in general hospitals but is associated with significant and severe psychiatric and medical comorbidities. Further research is needed into the optimal diagnosis, workup, and treatment of catatonia in pediatric patients.

## Introduction

Catatonia is a disorder characterized by a range of motor and behavioral disturbances which can be diagnosed in both pediatric and adult patients (1). Diagnosis is based on clinical exam (2), noting a series of motor, speech, and activity traits that may be observed or elicited (3). Pediatric catatonia and its manifestations are associated with strikingly high morbidity and mortality, with a 6,000% increased mortality relative to age-matched controls over a 4 year follow-up period (4). Benzodiazepines are the gold-standard treatment of catatonia (5), with electroconvulsive therapy for refractory cases and only weak evidence for alternative medications (6, 7). Catatonia has been diagnosed across the age spectrum, with a reported prevalence of catatonia of 5.5% among new intakes at a child and adolescent psychiatry outpatient clinic (8), and a rate of 17.8% among outpatients with disorders linked to catatonia (including autism, psychosis, and intellectual disability) (9).

While catatonia has been studied for over a century, and large-scale studies have investigated clinical factors associated with catatonia diagnosis in adults (10), there is relatively little research in the pediatric population. The largest existing cohort of pediatric catatonia patients is a multi-decade single-center study of 89 children and adolescents diagnosed with catatonia in France (11), and there is a paucity of multi-center studies. As a result, little is known about the demographics of pediatric patients diagnosed with catatonia nor of their treatment or comorbidities. This study explores the demographics of patients hospitalized for catatonia in general hospitals, as well as comorbid conditions and treatments using a national all-payors database of pediatric hospitalizations in the United States.

## Methods

### Data Source

Analysis used the 2019 version of the Kids' Inpatient Database (KID) from the Healthcare Cost and Utilization Project (HCUP) of the Agency for Healthcare Research and Quality. The KID samples 80% of non-newborn discharges (and 10% of normal newborn births) from 3,998 general hospitals in 49 states in the United States, regardless of payor. The sample is then weighted to provide national estimates. In total the 2019 KID contains a weighted 5,902,538 hospitalizations, including normal newborn births. As this is a de-identified, publicly-available database this study was determined to be Not Human Subjects Research by the MassGeneral Brigham Institutional Review Board.

### Data Collection

Individual hospitalizations in the KID can include up to 40 discharge diagnoses. Catatonia hospitalizations were those whose discharge diagnoses included the *International Statistical Classification of Diseases, Tenth Revision, Clinical Modification* (*ICD-10-CM*) codes F06.1 (catatonic disorder due to a known physiological condition) or F20.2 (catatonic schizophrenia). These limited nosologic categories prevent any fine-grained analysis of catatonic syndrome subtypes so our goal in this paper is to present data on what clinicians diagnosed as catatonia using these 2 *ICD-10-CM* categories and the associated diagnoses and comorbidities that were noted in this dataset. Hospitalizations were classified as primary catatonia if the primary discharge diagnosis was catatonia, while those with catatonia as a secondary diagnosis were classified as secondary catatonia. Comorbidities were identified from the discharge diagnoses, and classified according to the Pediatric Clinical Classification System (PECCS) (12), which groups the 72,446 *ICD-10-CM* diagnosis codes into 834 clinically distinctive categories for pediatric medical conditions.

### Statistical Analysis

The design of the KID incorporates survey design, with hospitals clustered within strata and then non-newborn discharges sampled 80% without replacement. Because of this survey design, all statistics from the sample have a corresponding variance. This variance is presented for the total number of patients in the sample, with other values presented as weighted point estimates. Baseline demographics of the primary catatonia and secondary catatonia groups were compared using the χ2 test for categorical variables. Age, hospital length of stay, and total cost were compared using the Mann-Whitney *U*-test. All tests were 2-sided, with *P*-values < 0.05 considered statistically significant, with no correction for multiple hypothesis testing. All analyses were conducted on data weighted according to the appropriate KID discharge weight to obtain national estimates. Analyses were conducted using SPSS (version 28; IBM Software, Inc, Armonk, NY). Results are reported in accordance with the REporting of studies Conducted using Observational Routinely-collected health Data (RECORD) statement (13).

## Results

**Figure 1:**
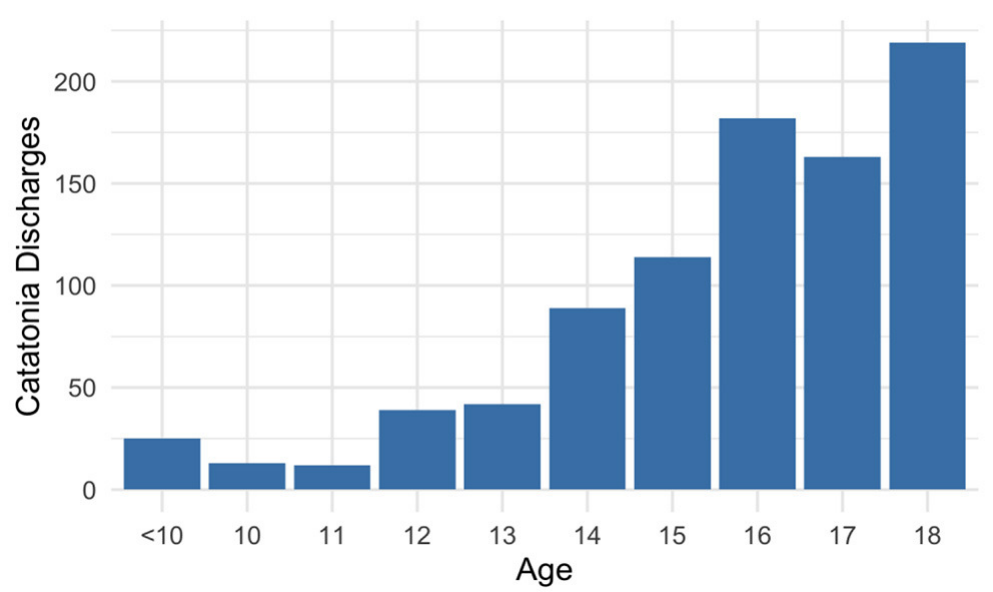
Age distribution of patients diagnosed with catatonia.

From the 2019 KID, 900 pediatric hospitalizations (95% CI: 850–949) included a discharge diagnosis of catatonia (Table 1). Of these 291 (95% CI: 276–306; 32.3%), had catatonia as the primary discharge diagnosis, while 609 (95% CI: 573–644; 67.7%) had catatonia as a secondary discharge diagnosis. Males (512) made up 56.9% of the sample. Mean age was 15.6 ± 2.6 years. The age distribution of patients was skewed toward older adolescents, but 25 patients (2.8%) were under 10 years old, and 89 (9.9%) were under age 13 (Figure 1). The racial breakdown of the sample was 308 (34.2%) White patients, 252 (28.0%) Black patients, 169 (18.8%) Hispanic patients, 56 (6.2%) Asian or Pacific Islander patients, and 70 (7.8%) other. Racial information was missing for 46 individuals (5.1%). Most hospitalizations occurred in urban regions, with 628 patients (69.8%) coming from counties with populations of >1 million individuals, and only 67 (7.4%) from counties with populations of <50,000. Medicaid was the primary payor for 466 patients (51.8%), with commercial insurance used for 390 (43.3%). Hospitalizations were non-elective for 786 (87.3%) patients, and 306 (34.0%) involved a transfer from another healthcare facility. At the end of hospitalization 674 (74.9%) were discharged home, while the remainder were transferred to another acute care hospital (54; 6.0%) or another healthcare facility (180; 20.0%). Median hospital length of stay was 9.0 days (IQR 5.0–21.0), with median healthcare charges of $48,457 (IQR $24,401–102,412).

**Table 1:** Baseline demographics.

	**Overall**		**Primary catatonia**		**Secondary catatonia**		
	***n***	**%**	***n***	**%**	***n***	**%**	
*n* (95% CI)	900 (850–949)		291 (276–306)		609 (573–644)		
Age (yrs; mean ± SD)	15.6 ± 2.6		15.9 ± 2.3		15.4 ± 2.7		*P* = 0.121
Male Sex	512	56.9%	194	66.7%	318	52.2%	*P* <0.0001
Race							*P* = 0.426
White	308	34.2%	91	31.3%	217	35.6%	
Black	252	28.0%	95	32.6%	157	25.8%	
Hispanic	169	18.8%	53	18.2%	116	19.0%	
Asian or Pacific Islander	56	6.2%	16	5.5%	40	6.6%	
Other	70	7.8%	23	7.9%	47	7.7%	
Missing	46	5.1%	14	4.8%	32	5.3%	
Hospital region							*P* = 0.089
Northeast	182	20.2%	53	18.2%	129	21.2%	
Midwest	230	25.6%	83	28.5%	147	24.1%	
South	293	32.6%	83	28.5%	210	34.5%	
West	194	21.6%	72	24.7%	122	20.0%	
Population of County of Residence							*P* = 0.380
Central metro county >1 million	376	41.8%	130	44.7%	246	40.4%	
Fringe metro county >1 million	252	28.0%	76	26.1%	176	28.9%	
Metro Area 250,000–999,999	134	14.9%	47	16.2%	87	14.3%	
Metro Area 50,000–249,000	63	7.0%	16	5.5%	47	7.7%	
Micropolitan	41	4.6%	13	4.5%	28	4.6%	
Non-core county	26	2.9%	<11	<3.8%	21	3.4%	
Household income quartile for Pt ZIP code							*P* = 0.153
1	224	24.9%	76	26.1%	148	24.3%	
2	229	25.4%	60	20.6%	169	27.8%	
3	235	26.1%	82	28.2%	153	25.1%	
4	198	22.0%	68	23.4%	130	21.3%	
Discharge quarter							*P* = 0.186
Jan–Mar	223	24.8%	83	28.5%	140	23.0%	
Apr–Jun	264	29.3%	81	27.8%	183	30.0%	
Jul–Sep	207	23.0%	58	19.9%	149	24.5%	
Oct–Dec	205	22.8%	70	24.1%	135	22.2%	
Admission Type							*P* = 0.009
Elective	112	12.4%	24	8.2%	88	14.4%	
Non-elective	786	87.3%	265	91.1%	521	85.6%	
Primary payor							*P* = 0.903
Medicaid	466	51.8%	148	50.9%	318	52.2%	
Commercial insurance	390	43.3%	129	44.3%	261	42.9%	
Other	42	4.7%	13	4.5%	29	4.8%	
Admission status							*P* = 0.118
Not transferred in	592	65.8%	204	70.1%	388	63.7%	
Transferred from acute care hospital	196	21.8%	58	19.9%	138	22.7%	
Transferred from another facility	110	12.2%	28	9.6%	82	13.5%	
Patient disposition							*P* = 0.460
Discharged	674	74.9%	221	75.9%	453	74.4%	
Transferred to acute care hospital	54	6.0%	19	6.5%	35	5.7%	
Transferred to another facility	180	20.0%	51	17.5%	129	21.2%	
Hospital length of stay (days, median, IQR)	9.0 (5.0–21.0)		9.0 (5.0–20.9)		9.0 (5.0–21.5)		*P* = 0.749
Total charges ($, median, IQR)	48,457 (24,401–102,412)		43,129 (22,879–87,183)		52,029 (25,309–121,791)		*P* = 0.009

*Statistical comparisons are between primary catatonia and secondary catatonia hospitalizations*.

Hospitalizations for primary catatonia involved male patients more often than did hospitalizations with secondary catatonia (66.7 vs. 52.2%; χ^2^ = 16.76, df = 1, *P* < 0.0001), and were less likely to involve elective admission (8.2 vs. 14.4%; χ^2^ = 6.780, df = 1, *P* = 0.009). Primary catatonia hospitalizations had overall lower hospital charges (median of $43,129 vs. $52,029; Mann-Whitney *U, U* = 42,901, *P* = 0.009). Primary and secondary catatonia discharges did not differ on other demographic or hospital factors including age, race, region, income, primary payor, or hospital length of stay (Table 1).

Primary discharge diagnoses for hospitalizations involving catatonia are listed in Table 2. Other than catatonia itself, psychiatric diagnoses include psychotic disorders (165; 19.3%), major depressive disorder (69; 7.7%), bipolar disorder (39; 4.3%) and substance-related disorders (20; 2.2%). Notably, developmental, neurologic, and autoimmune illnesses were also common. In total, encephalitis, autism spectrum disorders, other neurologic disorders (of which 13 of 18 are encephalopathy), and systemic lupus erythematosus were the principal diagnoses for 106 discharges (11.8%).

**Table 2:** Primary diagnostic categories for hospitalizations involving catatonia.

**Primary discharge diagnosis**	***n***	**%**
Catatonia	291	32.3%
Schizophrenia and other psychotic disorders	165	18.3%
Major depressive disorder	69	7.7%
Encephalitis	56	6.2%
Bipolar disorder	39	4.3%
Substance-related disorders	20	2.2%
Autistic disorder	18	2.0%
Other nervous system disorders	18	2.0%
Systemic lupus erythematosus and connective tissue disorders	14	1.6%
Transient alteration of awareness	13	1.4%
Dehydration	12	1.3%

*Included are all categories with ≥ 11 discharges. The category of “other nervous system disorders” includes 13 codes related to encephalitis, while “transient alteration of awareness” all codes are for “altered mental status, unspecified” (R4182)*.

As hospitalizations could include up to 40 overall diagnoses, individual hospitalizations could include diagnoses in multiple medical and psychiatric categories. Analyzing all diagnoses (up to 40 primary and secondary discharge diagnoses) for all 900 catatonia hospitalizations, the 12 most common diagnoses (excluding catatonia itself), each occurring in >10% of patients, include six psychiatric comorbidities: unspecified anxiety disorder (216; 24.0%), major depressive disorder single episode (137; 15.2%), suicidal ideation (124; 13.8%), unspecified psychosis (123; 13.7%), unspecified insomnia (115; 12.8%), and unspecified attention-deficit hyperactivity disorder (113; 12.6%). The most common individual medical diagnoses were unspecified constipation (125; 13.9%), dehydration (112; 12.4%), and unspecified tachycardia (91; 10.1%). Autism spectrum disorder was diagnosed in 142 (15.8%). A full table of codes diagnosed in ≥11 discharges is given in Supplementary Table 1.

Grouping individual billing codes into relevant pediatric categories using the PECCS demonstrates diverse comorbidities among patients diagnosed with catatonia. Table 3 lists the PECCS categories diagnosed >100 times and includes the top three individual billing codes contributing to the category. Consistent with the individual discharge diagnoses, PECCS categories are dominated by psychiatric comorbidities including psychotic disorders, anxiety disorders, substance use disorders, and mood disorders. Neurologic disorders are also common (251 diagnoses), as are autism spectrum disorders (142 diagnoses) and other developmental disorders (160 diagnoses). A full list of PECCS categories is given in Supplementary Table 2. While the KID cannot distinguish between complications of a diagnosis that occurred in-hospital vs. were present on admission, many of the discharge diagnoses are potential complications of catatonia. These include constipation (125), tachycardia (91), dehydration (112), the use of physical restraints (86), urinary incontinence (52), restlessness and agitation (50), hypokalemia (40), malnutrition (37), acute renal failure (35), and rhabdomyolysis (27).

**Table 3:** Discharge diagnoses for pediatric catatonia hospitalizations.

**Code**	***N***	**%**	**Description**
**659000**	**763**		**Schizophrenia and other psychotic disorders**
F202	452	50.2%	Catatonic schizophrenia
F29	123	13.7%	Unspecified psychosis not due to a substance or known physiological condition
F23	43	4.8%	Brief psychotic disorder
**259000**	**621**		**Residual codes; unclassified**
Z818	151	16.8%	Family history of other mental and behavioral disorders
Z9114	89	9.9%	Patient's other non-compliance with medication regimen
Z781	86	9.6%	Physical restraint status
**670000**	**513**		**Miscellaneous mental health disorders**
F061	456	50.7%	Catatonic disorder due to known physiological condition
**651000**	**327**		**Anxiety disorders**
F419	216	24.0%	Anxiety disorder, unspecified
F411	48	5.3%	Generalized anxiety disorder
F410	34	3.8%	Panic disorder (episodic paroxysmal anxiety)
**661000**	**257**		**Substance-related disorders**
F1290	66	7.3%	Cannabis use, unspecified, uncomplicated
F1210	43	4.8%	Cannabis abuse, uncomplicated
F17210	21	2.3%	Nicotine dependence, cigarettes, uncomplicated
**95000**	**251**		**Other nervous system disorders**
R4701	40	4.4%	Aphasia
G9340	35	3.9%	Encephalopathy, unspecified
G9349	30	3.3%	Other encephalopathy
**657003**	**248**		**Mood disorders (major depressive disorder)**
F329	137	15.2%	Major depressive disorder, single episode, unspecified
F333	31	3.4%	Major depressive disorder, recurrent, severe w psychotic symptoms
F323	30	3.3%	Major depressive disorder, single episode, severe w psychotic features
**662000**	**209**		**Suicide and intentional self-inflicted injury**
R45851	124	13.8%	Suicidal ideations
Z915	64	7.1%	Personal history of self-harm
**58000**	**190**		**Other nutritional; endocrine; and metabolic disorders**
R6250	42	4.7%	Unspecified lack of expected normal physiological development in childhood
R634	20	2.2%	Abnormal weight loss
E8339	18	2.0%	Other disorders of phosphorus metabolism
**255000**	**169**		**Administrative/social admission**
Z62810	35	3.9%	Personal history of physical and sexual abuse in childhood
Z590	13	1.4%	Homelessness
**257000**	**167**		**Other aftercare**
Z79899	116	12.9%	Other long term (current) drug therapy
Z7951	14	1.6%	Long term (current) use of inhaled steroids
Z7952	13	1.4%	Long term (current) use of systemic steroids
**654000**	**160**		**Developmental disorders**
F79	52	5.8%	Unspecified intellectual disabilities
F71	17	1.9%	Moderate intellectual disabilities
F70	15	1.7%	Mild intellectual disabilities
**253000**	**155**		**Allergic reactions**
Z888	38	4.2%	Allergy status to other drug/meds/biological substance status
Z880	33	3.7%	Allergy status to penicillin
Z91018	12	1.3%	Allergy to other foods
**155004**	**147**		**Constipation**
K5900	125	13.9%	Constipation, unspecified
K5909	13	1.4%	Other constipation
**655001**	**142**		**Autistic disorder**
F840	142	15.8%	Autistic disorder
**259001**	**136**		**Sleep disturbances**
G4700	115	12.8%	Insomnia, unspecified
G479	13	1.4%	Sleep disorder, unspecified
**652001**	**126**		**Attention-deficit hyperactivity disorder**
F909	113	12.6%	Attention-deficit hyperactivity disorder, unspecified type
**106000**	**122**		**Cardiac dysrhythmias**
R000	91	10.1%	Tachycardia, unspecified
R001	20	2.2%	Bradycardia, unspecified
**55001**	**117**		**Dehydration**
E860	112	12.4%	Dehydration
**128000**	**104**		**Asthma**
J45909	88	9.8%	Unspecified asthma, uncomplicated

*Included are all primary and secondary discharge diagnoses for all hospitalizations, so individual hospitalizations may include up to 40 of the below codes. Bolded rows are PECCS categories, and under each category are individual ICD-10-CM codes contributing to that category. The top 3 codes (diagnosed >10 times) are included for each category*.

In total 390 catatonia hospitalizations (43.3%) had at least one procedure performed during admission (Table 4). The most frequent procedure was a lumbar puncture, performed in 189 (21.0%) of hospitalizations. Electroconvulsive therapy was utilized in 48 admissions (5.3%). Supportive care procedures included central line insertion (39; 4.3%), enteral feeding (34; 3.8%), gastrostomy tube placement (22; 2.4%), and mechanical ventilation (19; 2.1%). Immunomodulatory therapy by intravenous globulin infusion (21; 2.3%) and plasmapheresis (19; 2.1%) were also performed.

**Table 4:** Top 10 procedures performed during hospitalization based on number of hospitalizations including the indicated procedure.

**Procedure**	***n***	**%**
Any procedure	390	43.3%
Lumbar puncture	189	21.0%
Electroencephalogram	77	8.6%
MRI brain	62	6.9%
Electroconvulsive therapy	48	5.3%
Central line insertion	39	4.3%
Enteral feeding	34	3.8%
Gastrostomy tube placement	22	2.4%
Intravenous globulin infusion	21	2.3%
Plasmapheresis	19	2.1%
Mechanical ventilation	19	2.1%

*Hospitalizations may have involved more than one application of each procedure*.

## Discussion

In 2019, a total of 900 discharges (95% CI: 850–949) of pediatric patients from general medical hospitals included a diagnosis of catatonia. These results add to understanding of the demographics and comorbidities of catatonia in pediatric patients.

Demographically, the incidence of pediatric catatonia increases with age, perhaps reflecting the increased diagnosis of serious mental illness in older teenagers (14). It is notable, however, that catatonia is diagnosed in younger children as well, and 9.9% of the hospitalizations in this sample are for pre-teens in whom the diagnosis may be less frequently considered. Given the ready treatment of catatonia with medications or electroconvulsive therapy (15), and the fact that longer length of catatonia correlates with greater treatment-resistance (16), a failure to diagnose the condition in younger patients subjects them to significant morbidity. As a result, it is important to consider the diagnosis in patients across the age spectrum. Males were diagnosed with catatonia more frequently than females, but our study did not find the 2:1 ratio of male to female patients demonstrated in one prior sample (17). Notably, females were overrepresented in cases of secondary catatonia, which may relate to higher rates of certain underlying illnesses, including paraneoplastic limbic encephalitidies, lupus, and other autoimmune disorders (18, 19).

Catatonia discharges occurred at similar rates across the year, and included patients of all income strata, although income data is based on ZIP code of residence and not on individual patient data. Racially, patients diagnosed with catatonia do not match the demographics of children and adolescents in the United States as a whole, with non-Hispanic White individuals representing 34.2% of catatonia diagnoses vs. 50% of the children under age 18, and Black individuals representing 28.0% of catatonia patients vs. 14% of the pediatric population (population figures from the 2019 American Community Survey 1-year estimates) (20). This may reflect racial bias, neighborhood factors, cumulative trauma, and perinatal complications that cumulatively have led to Black patients being more readily diagnosed with psychotic disorders compared to mood disorders (21, 22). Differences in diagnosis by race were not significantly different for primary vs. secondary catatonia, however, so other factors may be responsible for the increased diagnosis of catatonia in children from racial minorities. Catatonia hospitalizations were overall costly, with a median hospital charge of $48,457 (IQR $24,401–102,412). This makes catatonia among the most costly pediatric mental health disorders on a per-case basis, with a 2009 analysis of the KID finding the most expensive pediatric mental health condition to be eating disorders, with a mean charge of $46,130 per hospitalization (23). In contrast hospitalizations for depression averaged $13,200 per case and psychosis $19,676, although these charges likely increased in the intervening decade.

Diagnostically, pediatric catatonia patients were more likely to have a primary psychiatric diagnosis of psychotic disorders (18.3%) than major depressive disorder (7.7%) or bipolar disorders (4.3%). Neurologic, developmental, and inflammatory illnesses were also common (11.8%). This diagnostic distribution has similarities with descriptions from prior pediatric catatonia literature. For instance, in a retrospective study of 52 child and adolescent psychiatric inpatients diagnosed with catatonia (mean age 16.8 years), psychotic disorders were diagnosed in 78.8%, affective disorders in 9.6%, and general medical illnesses (including epilepsy and systemic lupus erythematosus) in 15.4% (24). Similarly, a prospective series of 58 child and adolescent psychiatric inpatients diagnosed with catatonia (mean age 15.0) found schizophrenia as the main diagnosis in 55.2%, major depression in 31.0%, and mania in 10.3% of patients (17). Additionally, post-traumatic stress disorder was diagnosed in 58 patients in this sample (6.4%). Acute trauma has been linked to the development of catatonia (25), with one theory positing catatonia as an exaggerated form of fear response (1, 26), but the nature and time course of trauma in these patients is not systematically captured by the KID. This sample likewise demonstrated a high proportion of medical illnesses, with 22.4% diagnosed with conditions including anti-NMDA receptor encephalitis, systemic lupus erythematosus, and epilepsy.

Notably, this distribution of primary psychiatric diagnoses is different from the spectrum of comorbidity seen in adult patients, in which higher proportions of mood disorders (particularly bipolar disorder) than psychotic disorders have been found in catatonic patients. For instance, in a prospective evaluation of 55 adult inpatients with catatonia, Abrams and Taylor found only 7.3% to have schizophrenia while 62% had mania and 9% had depression (27). A further study of 201 adult psychiatric inpatients found a catatonia prevalence of 10.5% in psychosis vs. 12.8% in bipolar disorder and 7.1% in unipolar depression (28).

Analysis of comorbidities reveals a high rate of comorbid medical and psychiatric illnesses among pediatric patients diagnosed with catatonia. In addition to the psychotic and affective disorders discussed above, there were 327 comorbid anxiety disorders and 257 comorbid substance use disorders. Notably, anxiety disorders are not commonly represented among causes of catatonia for adults, though catatonia is believed by some to be an evolutionary fear response to acute stress (16). Substance use disorder diagnoses were mostly related to cannabis, which is the most commonly abused non-alcohol substance in adolescents (29). Interestingly, while cannabis and particularly synthetic cannabinoids are linked to development or worsening of psychosis (30), cannabis use has not been highly associated with catatonia in adults. Developmental disorders were also very common, with 160 diagnoses of developmental disorders and 142 of autism spectrum disorder. The connection between ASD and catatonia is particularly important for clinicians to recognize, as some estimates suggest that 4–17% of patients with ASD will have an episode of catatonia in adolescence or adulthood (31). Furthermore, the diverse nature of medical diagnoses that may be complications of catatonia highlights the substantial clinical impact of catatonia on multiple organ systems.

Diagnostic and therapeutic procedures were commonly performed during hospitalizations for catatonia, with 390 (43.3%) involving at least one procedure performed during the hospitalization. Diagnostically, lumbar punctures were more common than either electroencephalography or brain imaging. As there is no clear role for lumbar puncture in the diagnosis of catatonia itself, these procedures were likely performed to rule out neurologic or medical causes of catatonia such as autoimmune encephalitis (32), although the recommended workup for this condition generally involves lumbar puncture, EEG, and MRI. Beyond diagnostic procedures, a significant fraction of patients required invasive treatments. This includes electroconvulsive therapy, which is an FDA-approved device for the treatment for catatonia in patients aged 13 or older, for 48 patients (5.3%). This is a lower rate of ECT utilization than in adult patients, with a nationwide inpatient survey finding 8.3% of adult patients diagnosed with catatonia received ECT (33). As the efficacy cognitive side effects of ECT are comparable in adolescents and young adults (34), this may represent an underutilization of this treatment. Pediatric access to ECT is curtailed by law in 21 states (35), and nationwide children make up <1% of ECT recipients (36), but this sample demonstrates that it remains a necessary treatment for some catatonic children. Furthermore, many of the patients in the sample required significant supportive treatments that could only be provided in a general medical hospital including enteral feeding, central lines, and mechanical ventilation.

Strengths of this study derive from utilizing nationwide all-payor database that includes 80% of pediatric discharges from general medical hospitals. This detailed capture of discharges allows for the accurate measure of rare comorbidities and provides unbiased estimates of clinical practice throughout the US. This also allows for a very large sample size—indeed the 900 discharges described here are an order of magnitude larger than the previous largest pediatric catatonia cohort (89 patients admitted to a single center over 20 years) (11). As a result, the demographic information reported here likely provides the most comprehensive demographic accounting of the condition yet reported.

Limitations of the study derive from the retrospective observational nature of the sample. Cases are derived from billing data, and so to be counted as a pediatric catatonia case in this study requires that the treating team accurately diagnosed the condition. While there are no studies on the accuracy of pediatric provider identification of catatonia, among adult physicians in the hospital setting, one retrospective chart review by expert clinicians found that only 59% of likely catatonia cases were diagnosed by the treatment team (37), with neurologists rarely identifying catatonia in the emergency department (38). As a result this study may underestimate the true number of catatonia cases as catatonic patients who were not diagnosed with the condition by the treatment team would erroneously be counted as non-cases. Conversely, it is unlikely catatonia will be listed as a discharge diagnosis unless it was clearly diagnosed, so the hospitalizations identified here likely represent true cases. Moreover, the KID only includes general hospitals and does not survey freestanding psychiatric hospitals or long-term rehabilitation hospitals; the incidence of pediatric catatonia in those settings is unclear. Additionally, the 2019 KID utilizes *ICD-10-CM* diagnostic codes. There are significant differences in the classification of catatonia among diagnostic schema, and for instance *ICD-11* introduces substantial revisions to catatonia diagnosis (39). As the KID only contains *ICD-10-CM* codes, we are unable to assess if diagnoses might be different using alternative classification schemes. Finally, since the KID weights discharges and not individual patients, some individuals who may have been hospitalized more than once for catatonia (or in more than one general hospital for the same episode) may be counted multiple times.

## Data Availability Statement

Publicly available datasets were analyzed in this study. This data can be found here: The dataset analyzed for this study can be obtained from the Healthcare Cost and Utilization Project (HCUP), Agency for Healthcare Research and Quality at: https://www.hcup-us.ahrq.gov/db/nation/kid/kiddbdocumentation.jsp.

## Author Contributions

JL, MK, CF-R, GF, and SB contributed to conception and design of the study. JL and MK performed the statistical analysis. JL wrote the first draft of the manuscript. MK, CF-R, GF, and SB wrote sections of the manuscript. All authors contributed to manuscript revision, read, and approved the submitted version. All authors agree to be accountable for the content of the work.

## Funding

This work was supported by the National Institute of Mental Health (R25MH094612), JL and the Avery D. Weisman Fund of the Massachusetts General Hospital Department of Psychiatry.

## Conflict of Interest

MK is currently employed by and has equity in Watershed Informatics, whose work is unrelated to pediatric catatonia. The remaining authors declare that the research was conducted in the absence of any commercial or financial relationships that could be construed as a potential conflict of interest.

## Publisher's Note

All claims expressed in this article are solely those of the authors and do not necessarily represent those of their affiliated organizations, or those of the publisher, the editors and the reviewers. Any product that may be evaluated in this article, or claim that may be made by its manufacturer, is not guaranteed or endorsed by the publisher.
