# Supplementary material for: The Incidence of Catatonia Diagnosis Among Pediatric Patients Discharged From General Hospitals in the United States: A Kids' Inpatient Database Study
Source: Front Psychiatry. 2022 Apr 29;13:878173. doi: 10.3389/fpsyt.2022.878173 (PMC9106281; doi:10.3389/fpsyt.2022.878173)
Supplement: Supplementary file 1 [file Table_1.DOCX]

Supplementary Material

# Supplementary Tables

Supplemental Table S1: *ICD-10-CM* codes diagnosed at discharge ≥ 11 times among the 900 hospitalizations involving catatonia. Included are all primary and secondary diagnoses (with each discharge including up to 40 diagnoses). Diagnostic codes listed in descending order of frequency, along with the associated PECCS code and category for each *ICD-10-CM* code.

| Code | Description | N | Percent | PECCS Code | PECCS Category |
| --- | --- | --- | --- | --- | --- |
| F061 | Catatonic disorder due to known physiological condition | 456 | 50.7% | 670000 | Miscellaneous mental health disorders |
| F202 | Catatonic schizophrenia | 452 | 50.2% | 659000 | Schizophrenia and other psychotic disorders |
| F419 | Anxiety disorder, unspecified | 216 | 24.0% | 651000 | Anxiety disorders |
| Z818 | Family history of other mental and behavioral disorders | 151 | 16.8% | 259000 | Residual codes; unclassified |
| F840 | Autistic disorder | 142 | 15.8% | 655001 | Autistic disorder |
| F329 | Major depressive disorder, single episode, unspecified | 137 | 15.2% | 657003 | Mood disorders (major depressive disorder) |
| K5900 | Constipation, unspecified | 125 | 13.9% | 155004 | Constipation |
| R45851 | Suicidal ideations | 124 | 13.8% | 662000 | Suicide and intentional self-inflicted injury |
| F29 | Unsp psychosis not due to a substance or known physiol cond | 123 | 13.7% | 659000 | Schizophrenia and other psychotic disorders |
| Z79899 | Other long term (current) drug therapy | 116 | 12.9% | 257000 | Other aftercare |
| G4700 | Insomnia, unspecified | 115 | 12.8% | 259001 | Sleep disturbances |
| F909 | Attention-deficit hyperactivity disorder, unspecified type | 113 | 12.6% | 652001 | Attention-deficit hyperactivity disorder |
| E860 | Dehydration | 112 | 12.4% | 55001 | Dehydration |
| R000 | Tachycardia, unspecified | 91 | 10.1% | 106000 | Cardiac dysrhythmias |
| Z9114 | Patient's other noncompliance with medication regimen | 89 | 9.9% | 259000 | Residual codes; unclassified |
| J45909 | Unspecified asthma, uncomplicated | 88 | 9.8% | 128000 | Asthma |
| Z781 | Physical restraint status | 86 | 9.6% | 259000 | Residual codes; unclassified |
| G0481 | Other encephalitis and encephalomyelitis | 69 | 7.7% | 77000 | Encephalitis (except that caused by tuberculosis or sexually transmitted disease) |
| F1290 | Cannabis use, unspecified, uncomplicated | 66 | 7.3% | 661000 | Substance-related disorders |
| I10 | Essential (primary) hypertension | 64 | 7.1% | 98000 | Essential hypertension |
| Z915 | Personal history of self-harm | 64 | 7.1% | 662000 | Suicide and intentional self-inflicted injury |
| F4310 | Post-traumatic stress disorder, unspecified | 58 | 6.4% | 651001 | Posttraumatic stress disorder |
| F940 | Selective mutism | 54 | 6.0% | 655000 | Disorders usually diagnosed in infancy childhood or adolescence |
| F79 | Unspecified intellectual disabilities | 52 | 5.8% | 654000 | Developmental disorders |
| R32 | Unspecified urinary incontinence | 52 | 5.8% | 163000 | Genitourinary symptoms and ill-defined conditions |
| F429 | Obsessive-compulsive disorder, unspecified | 51 | 5.7% | 651003 | OCD |
| E559 | Vitamin D deficiency, unspecified | 50 | 5.6% | 52002 | Vitamin deficiency |
| R451 | Restlessness and agitation | 50 | 5.6% | 259000 | Residual codes; unclassified |
| F319 | Bipolar disorder, unspecified | 49 | 5.4% | 657002 | Mood disorders (bipolar disorder) |
| R4182 | Altered mental status, unspecified | 49 | 5.4% | 259003 | Transient alteration of awareness |
| F411 | Generalized anxiety disorder | 48 | 5.3% | 651000 | Anxiety disorders |
| F1210 | Cannabis abuse, uncomplicated | 43 | 4.8% | 661000 | Substance-related disorders |
| F23 | Brief psychotic disorder | 43 | 4.8% | 659000 | Schizophrenia and other psychotic disorders |
| N390 | Urinary tract infection, site not specified | 42 | 4.7% | 159000 | Urinary tract infections |
| R6250 | Unsp lack of expected normal physiol dev in childhood | 42 | 4.7% | 58000 | Other nutritional; endocrine; and metabolic disorders |
| E876 | Hypokalemia | 40 | 4.4% | 55000 | Fluid and electrolyte disorders |
| F2081 | Schizophreniform disorder | 40 | 4.4% | 659000 | Schizophrenia and other psychotic disorders |
| R4701 | Aphasia | 40 | 4.4% | 95000 | Other nervous system disorders |
| Z888 | Allergy status to oth drug/meds/biol subst status | 38 | 4.2% | 253000 | Allergic reactions |
| E43 | Unspecified severe protein-calorie malnutrition | 37 | 4.1% | 52001 | Malnutrition |
| K219 | Gastro-esophageal reflux disease without esophagitis | 36 | 4.0% | 138002 | Gastroesophageal reflux and esophagitis |
| Z9119 | Patient's noncompliance w oth medical treatment and regimen | 36 | 4.0% | 259000 | Residual codes; unclassified |
| G9340 | Encephalopathy, unspecified | 35 | 3.9% | 95000 | Other nervous system disorders |
| N179 | Acute kidney failure, unspecified | 35 | 3.9% | 157000 | Acute and unspecified renal failure |
| R569 | Unspecified convulsions | 35 | 3.9% | 83007 | Other convulsions |
| Z62810 | Personal history of physical and sexual abuse in childhood | 35 | 3.9% | 255000 | Administrative/social admission |
| F410 | Panic disorder [episodic paroxysmal anxiety] | 34 | 3.8% | 651000 | Anxiety disorders |
| G40909 | Epilepsy, unsp, not intractable, without status epilepticus | 34 | 3.8% | 83001 | Seizures w and w/o intractable epilepsy |
| Z880 | Allergy status to penicillin | 33 | 3.7% | 253000 | Allergic reactions |
| F333 | Major depressv disorder, recurrent, severe w psych symptoms | 31 | 3.4% | 657003 | Mood disorders (major depressive disorder) |
| F323 | Major depressv disord, single epsd, severe w psych features | 30 | 3.3% | 657003 | Mood disorders (major depressive disorder) |
| G9349 | Other encephalopathy | 30 | 3.3% | 95000 | Other nervous system disorders |
| F39 | Unspecified mood [affective] disorder | 27 | 3.0% | 657000 | Mood disorders |
| M6282 | Rhabdomyolysis | 27 | 3.0% | 211000 | Other connective tissue disease |
| R45850 | Homicidal ideations | 26 | 2.9% | 656000 | Impulse control disorders NEC |
| F22 | Delusional disorders | 25 | 2.8% | 659000 | Schizophrenia and other psychotic disorders |
| E669 | Obesity, unspecified | 24 | 2.7% | 58004 | Obesity |
| R633 | Feeding difficulties | 24 | 2.7% | 908 | Feeding difficulties and mismanagement |
| D509 | Iron deficiency anemia, unspecified | 23 | 2.6% | 59001 | Iron deficiency anemia |
| R1310 | Dysphagia, unspecified | 23 | 2.6% | 155009 | Dysphagia |
| R339 | Retention of urine, unspecified | 23 | 2.6% | 163000 | Genitourinary symptoms and ill-defined conditions |
| T43595A | Adverse effect of oth antipsychotics and neuroleptics, init | 23 | 2.6% | 2617 | Adverse effects of medical drugs |
| F200 | Paranoid schizophrenia | 22 | 2.4% | 659000 | Schizophrenia and other psychotic disorders |
| E871 | Hypo-osmolality and hyponatremia | 21 | 2.3% | 55003 | Hypoosmolality and/or hyponatremia |
| E872 | Acidosis | 21 | 2.3% | 55000 | Fluid and electrolyte disorders |
| F17210 | Nicotine dependence, cigarettes, uncomplicated | 21 | 2.3% | 661000 | Substance-related disorders |
| R51 | Headache | 21 | 2.3% | 84000 | Headache; including migraine |
| R001 | Bradycardia, unspecified | 20 | 2.2% | 106000 | Cardiac dysrhythmias |
| R634 | Abnormal weight loss | 20 | 2.2% | 58000 | Other nutritional; endocrine; and metabolic disorders |
| E162 | Hypoglycemia, unspecified | 19 | 2.1% | 51001 | Hypoglycemia |
| E46 | Unspecified protein-calorie malnutrition | 19 | 2.1% | 52001 | Malnutrition |
| F332 | Major depressv disorder, recurrent severe w/o psych features | 19 | 2.1% | 657003 | Mood disorders (major depressive disorder) |
| Z8249 | Family hx of ischem heart dis and oth dis of the circ sys | 19 | 2.1% | 259000 | Residual codes; unclassified |
| E8339 | Other disorders of phosphorus metabolism | 18 | 2.0% | 58000 | Other nutritional; endocrine; and metabolic disorders |
| G249 | Dystonia, unspecified | 18 | 2.0% | 81000 | Other hereditary and degenerative nervous system conditions |
| R509 | Fever, unspecified | 18 | 2.0% | 246000 | Fever of unknown origin |
| Z6851 | BMI pediatric, less than 5th percentile for age | 18 | 2.0% | 58000 | Other nutritional; endocrine; and metabolic disorders |
| Z87820 | Personal history of traumatic brain injury | 18 | 2.0% | 233000 | Intracranial injury |
| F71 | Moderate intellectual disabilities | 17 | 1.9% | 654000 | Developmental disorders |
| Q211 | Atrial septal defect | 17 | 1.9% | 213012 | Ostium secundum atrial septal defect |
| Z6852 | BMI pediatric, 5th percentile to less than 85% for age | 17 | 1.9% | 259000 | Residual codes; unclassified |
| F913 | Oppositional defiant disorder | 16 | 1.8% | 652004 | Oppositional defiant disorder |
| R1110 | Vomiting, unspecified | 16 | 1.8% | 250000 | Nausea and vomiting |
| R630 | Anorexia | 16 | 1.8% | 58000 | Other nutritional; endocrine; and metabolic disorders |
| Z6854 | BMI pediatric, greater than or equal to 95% for age | 16 | 1.8% | 58000 | Other nutritional; endocrine; and metabolic disorders |
| E440 | Moderate protein-calorie malnutrition | 15 | 1.7% | 52001 | Malnutrition |
| F05 | Delirium due to known physiological condition | 15 | 1.7% | 653000 | Delirium dementia and amnestic and other cognitive disorders |
| F1220 | Cannabis dependence, uncomplicated | 15 | 1.7% | 661000 | Substance-related disorders |
| F209 | Schizophrenia, unspecified | 15 | 1.7% | 659000 | Schizophrenia and other psychotic disorders |
| F312 | Bipolar disord, crnt episode manic severe w psych features | 15 | 1.7% | 657002 | Mood disorders (bipolar disorder) |
| F70 | Mild intellectual disabilities | 15 | 1.7% | 654000 | Developmental disorders |
| I959 | Hypotension, unspecified | 15 | 1.7% | 117000 | Other circulatory disease |
| R112 | Nausea with vomiting, unspecified | 15 | 1.7% | 250000 | Nausea and vomiting |
| R440 | Auditory hallucinations | 15 | 1.7% | 259000 | Residual codes; unclassified |
| R740 | Nonspec elev of levels of transamns & lactic acid dehydrgnse | 15 | 1.7% | 151000 | Other liver diseases |
| Z833 | Family history of diabetes mellitus | 15 | 1.7% | 259000 | Residual codes; unclassified |
| B9620 | Unsp Escherichia coli as the cause of diseases classd elswhr | 14 | 1.6% | 3000 | Bacterial infection; unspecified site |
| F88 | Other disorders of psychological development | 14 | 1.6% | 654000 | Developmental disorders |
| I4581 | Long QT syndrome | 14 | 1.6% | 105000 | Conduction disorders |
| R030 | Elevated blood-pressure reading, w/o diagnosis of htn | 14 | 1.6% | 117000 | Other circulatory disease |
| Z7951 | Long term (current) use of inhaled steroids | 14 | 1.6% | 257000 | Other aftercare |
| Z931 | Gastrostomy status | 14 | 1.6% | 155000 | Other gastrointestinal disorders |
| B370 | Candidal stomatitis | 13 | 1.4% | 4000 | Mycoses |
| D8989 | Oth disrd involving the immune mechanism, NEC | 13 | 1.4% | 57000 | Immunity disorders |
| E441 | Mild protein-calorie malnutrition | 13 | 1.4% | 52001 | Malnutrition |
| E870 | Hyperosmolality and hypernatremia | 13 | 1.4% | 55002 | Hyperosmolality and/or hypernatremia |
| F819 | Developmental disorder of scholastic skills, unspecified | 13 | 1.4% | 654000 | Developmental disorders |
| G40409 | Oth generalized epilepsy, not intractable, w/o stat epi | 13 | 1.4% | 83001 | Seizures w and w/o intractable epilepsy |
| G479 | Sleep disorder, unspecified | 13 | 1.4% | 259001 | Sleep disturbances |
| J690 | Pneumonitis due to inhalation of food and vomit | 13 | 1.4% | 129000 | Aspiration pneumonitis; food/vomitus |
| J9601 | Acute respiratory failure with hypoxia | 13 | 1.4% | 131000 | Respiratory failure; insufficiency; arrest |
| K5909 | Other constipation | 13 | 1.4% | 155004 | Constipation |
| M3219 | Oth organ or system involv in systemic lupus erythematosus | 13 | 1.4% | 210000 | Systemic lupus erythematosus and connective tissue disorders |
| Z590 | Homelessness | 13 | 1.4% | 255000 | Administrative/social admission |
| Z7952 | Long term (current) use of systemic steroids | 13 | 1.4% | 257000 | Other aftercare |
| F17200 | Nicotine dependence, unspecified, uncomplicated | 12 | 1.3% | 661000 | Substance-related disorders |
| F203 | Undifferentiated schizophrenia | 12 | 1.3% | 659000 | Schizophrenia and other psychotic disorders |
| F322 | Major depressv disord, single epsd, sev w/o psych features | 12 | 1.3% | 657003 | Mood disorders (major depressive disorder) |
| F418 | Other specified anxiety disorders | 12 | 1.3% | 651000 | Anxiety disorders |
| G259 | Extrapyramidal and movement disorder, unspecified | 12 | 1.3% | 81000 | Other hereditary and degenerative nervous system conditions |
| G43909 | Migraine, unsp, not intractable, without status migrainosus | 12 | 1.3% | 84000 | Headache; including migraine |
| G901 | Familial dysautonomia [Riley-Day] | 12 | 1.3% | 216000 | Nervous system congenital anomalies |
| R21 | Rash and other nonspecific skin eruption | 12 | 1.3% | 200006 | Rashes |
| R251 | Tremor, unspecified | 12 | 1.3% | 95000 | Other nervous system disorders |
| Z91018 | Allergy to other foods | 12 | 1.3% | 253000 | Allergic reactions |
| D72829 | Elevated white blood cell count, unspecified | 11 | 1.2% | 63000 | Diseases of white blood cells |
| F12159 | Cannabis abuse with psychotic disorder, unspecified | 11 | 1.2% | 661000 | Substance-related disorders |
| F809 | Developmental disorder of speech and language, unspecified | 11 | 1.2% | 654000 | Developmental disorders |
| L709 | Acne, unspecified | 11 | 1.2% | 200000 | Other skin disorders |
| R441 | Visual hallucinations | 11 | 1.2% | 89000 | Blindness and vision defects |
| R748 | Abnormal levels of other serum enzymes | 11 | 1.2% | 151000 | Other liver diseases |
| T380X5A | Adverse effect of glucocort/synth analog, init | 11 | 1.2% | 2617 | Adverse effects of medical drugs |

Supplemental Table S2: PECCS diagnostic categories diagnosed ≥ 11 times among the primary and secondary discharge diagnoses (up to 40 diagnoses per discharge) for the 900 catatonia hospitalizations.

| PECCS Category | Description | n |
| --- | --- | --- |
| 659000 | Schizophrenia and other psychotic disorders | 763 |
| 259000 | Residual codes; unclassified | 621 |
| 670000 | Miscellaneous mental health disorders | 513 |
| 651000 | Anxiety disorders | 327 |
| 661000 | Substance-related disorders | 257 |
| 95000 | Other nervous system disorders | 251 |
| 657003 | Mood disorders (major depressive disorder) | 248 |
| 662000 | Suicide and intentional self-inflicted injury | 209 |
| 58000 | Other nutritional; endocrine; and metabolic disorders | 190 |
| 255000 | Administrative/social admission | 169 |
| 257000 | Other aftercare | 167 |
| 654000 | Developmental disorders | 160 |
| 253000 | Allergic reactions | 155 |
| 155004 | Constipation | 147 |
| 655001 | Autistic disorder | 142 |
| 259001 | Sleep disturbances | 136 |
| 652001 | Attention-deficit hyperactivity disorder | 126 |
| 106000 | Cardiac dysrhythmias | 122 |
| 55001 | Dehydration | 117 |
| 128000 | Asthma | 104 |
| 163000 | Genitourinary symptoms and ill-defined conditions | 99 |
| 657002 | Mood disorders (bipolar disorder) | 91 |
| 52001 | Malnutrition | 84 |
| 2617 | Adverse effects of medical drugs | 81 |
| 81000 | Other hereditary and degenerative nervous system conditions | 81 |
| 655000 | Disorders usually diagnosed in infancy childhood or adolescence | 80 |
| 55000 | Fluid and electrolyte disorders | 77 |
| 77000 | Encephalitis (except that caused by tuberculosis or sexually transmitted disease) | 77 |
| 98000 | Essential hypertension | 64 |
| 651001 | Posttraumatic stress disorder | 63 |
| 83001 | Seizures w and w/o intractable epilepsy | 57 |
| 259003 | Transient alteration of awareness | 55 |
| 52002 | Vitamin deficiency | 54 |
| 117000 | Other circulatory disease | 52 |
| 651003 | OCD | 52 |
| 159000 | Urinary tract infections | 48 |
| 211000 | Other connective tissue disease | 48 |
| 2621 | External cause codes: place of occurrence | 41 |
| 84000 | Headache; including migraine | 38 |
| 138002 | Gastroesophageal reflux and esophagitis | 37 |
| 657000 | Mood disorders | 37 |
| 157000 | Acute and unspecified renal failure | 35 |
| 58004 | Obesity | 35 |
| 83007 | Other convulsions | 35 |
| 3000 | Bacterial infection; unspecified site | 33 |
| 85000 | Coma; stupor; and brain damage | 33 |
| 151000 | Other liver diseases | 32 |
| 250000 | Nausea and vomiting | 32 |
| 656000 | Impulse control disorders NEC | 31 |
| 244000 | Other injuries and conditions due to external causes | 30 |
| 200000 | Other skin disorders | 28 |
| 155009 | Dysphagia | 27 |
| 210000 | Systemic lupus erythematosus and connective tissue disorders | 27 |
| 48000 | Thyroid disorders | 27 |
| 652002 | Conduct disorder | 27 |
| 4000 | Mycoses | 26 |
| 155000 | Other gastrointestinal disorders | 24 |
| 89000 | Blindness and vision defects | 24 |
| 908 | Feeding difficulties and mismanagement | 24 |
| 131000 | Respiratory failure; insufficiency; arrest | 23 |
| 181000 | Other complications of pregnancy | 23 |
| 233000 | Intracranial injury | 23 |
| 59001 | Iron deficiency anemia | 23 |
| 653000 | Delirium dementia and amnestic and other cognitive disorders | 23 |
| 55003 | Hypoosmolality and/or hyponatremia | 21 |
| 105000 | Conduction disorders | 20 |
| 246000 | Fever of unknown origin | 20 |
| 650000 | Adjustment disorders | 20 |
| 82001 | Cerebral palsy | 20 |
| 51001 | Hypoglycemia | 19 |
| 216000 | Nervous system congenital anomalies | 18 |
| 217000 | Other congenital anomalies | 18 |
| 57000 | Immunity disorders | 18 |
| 663000 | Screening and history of mental health and substance abuse codes | 18 |
| 91000 | Other eye disorders | 18 |
| 118000 | Phlebitis; thrombophlebitis and thromboembolism | 17 |
| 213012 | Ostium secundum atrial septal defect | 17 |
| 258000 | Other screening for suspected conditions (not mental disorders or infectious disease) | 17 |
| 59007 | Anemia unspecified | 17 |
| 658000 | Personality disorders | 17 |
| 652004 | Oppositional defiant disorder | 16 |
| 133000 | Other lower respiratory disease | 15 |
| 51000 | Other endocrine disorders | 15 |
| 10000 | Immunizations and screening for infectious disease | 14 |
| 171000 | Menstrual disorders | 14 |
| 238000 | Complications of surgical procedures or medical care | 14 |
| 251000 | Abdominal pain | 14 |
| 670001 | Conversion disorder | 14 |
| 7000 | Viral infection | 14 |
| 129000 | Aspiration pneumonitis; food/vomitus | 13 |
| 134001 | Allergic rhinitis | 13 |
| 198000 | Other inflammatory condition of skin | 13 |
| 2620 | External cause codes: unspecified | 13 |
| 55002 | Hyperosmolality and/or hypernatremia | 13 |
| 63000 | Diseases of white blood cells | 13 |
| 2000 | Septicemia (except in labor) | 12 |
| 200006 | Rashes | 12 |
| 204000 | Other non-traumatic joint disorders | 12 |
| 205000 | Spondylosis; intervertebral disc disorders; other back problems | 12 |
| 901 | Cellulitis | 12 |
| 904 | Scoliosis | 12 |
| 910 | Pneumonia | 12 |
| 94003 | Hearing loss | 12 |
| 252000 | Malaise and fatigue | 11 |
| 2603 | External cause codes: fall | 11 |
| 2611 | External cause codes: natural/environment | 11 |
| 2616 | Adverse effects of medical care | 11 |
